# Activity Engagement Patterns Among Older Chinese Immigrants

**DOI:** 10.1093/geroni/igab046.889

**Published:** 2021-12-17

**Authors:** Fengyan Tang, Mary Rauktis

**Affiliations:** University of Pittsburgh, Pittsburgh, Pennsylvania, United States

## Abstract

Activity engagement is a major component of well-being in later life. However, very few studies have focused on older immigrants who are often at risk for social isolation and psychological distress. We aim to map the pattern of activity engagement and examine its variations in relation to immigration-related factors and social aspects of neighborhoods in a representative sample of older Chinese immigrants. We used data from the Population Study of Chinese Elderly in Chicago (PINE), a population-based epidemiological study of US Chinese older adults that were conducted between 2011 and 2013 (N=3,157). Latent class analysis and multinominal regression analysis were conducted to identify activity engagement patterns and examine the associated factors. Four patterns of activity engagement were identified: restricted (15%), diverse (31%), informal social (32%), and community-based social (21%). Acculturation and family-oriented immigration differentiated the restricted from the diverse class membership. Positive attributes of social environment measured by social network size, positive social support, neighborhood cohesion, and sense of community were associated with the probabilities of class membership relative to the restricted class. Findings point to the importance of positive attributes of social environment in enhancing engagement with life among older Chinese immigrants. Efforts are needed to assist the vulnerable restricted group and recent older immigrants while meeting the demands of older immigrants who are less educated and less acculturated. Creating a supportive environment is important to provide information, access, and resources needed for activity engagement in the marginalized minority aging populations

